# Refinement of Protein Extraction Protocols for Human Peripheral Nerve Tissue

**DOI:** 10.1021/acsomega.4c11373

**Published:** 2025-02-02

**Authors:** Drifa Frostadottir, Charlotte Welinder, Raquel Perez, Lars B. Dahlin

**Affiliations:** a Department of Translational Medicine − Hand Surgery, Lund University, Malmö S-20502, Sweden; b Department of Hand Surgery, Skane University Hospital, Malmö S-20502, Sweden; c Faculty of Medicine, Department of Clinical Sciences Lund, Mass Spectrometry, Lund University, Lund S-20502, Sweden; d Unit for Social Epidemiology, Department of Clinical Sciences Malmö, Lund University, Malmö S-20502, Sweden; e Department of Biomedical and Clinical Sciences, Linköping University, SE-581 83 Linköping, Sweden

## Abstract

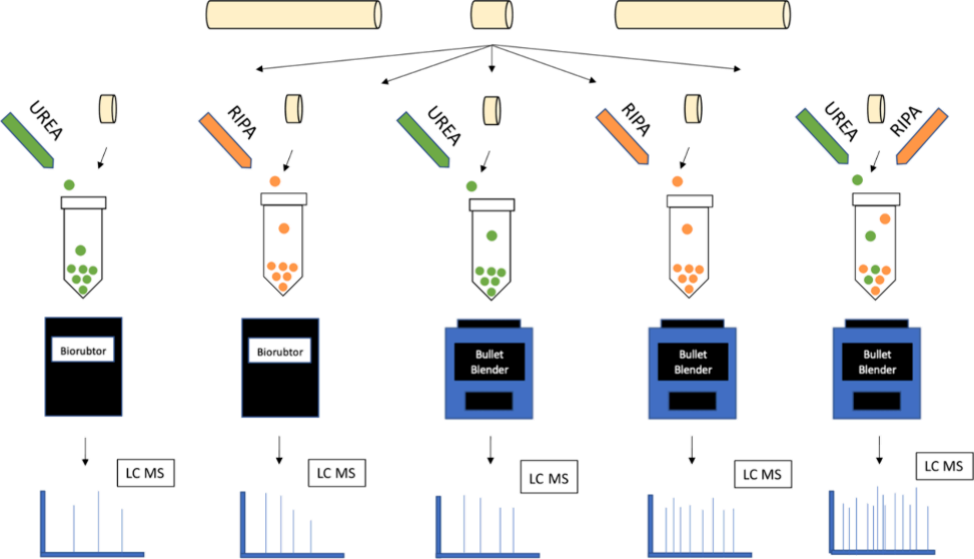

Our aim was to establish an effective method for protein extraction from freshly frozen human peripheral nerves, determine the minimum amount required for consistent protein extraction outcomes, and assess which method produced the highest number of protein identities. Five extraction methods were compared using 8 M urea and Ripa buffer using either the Bullet Blender or Bioruptor. Out of the total 2619 identified proteins, protein extraction using the Ripa buffer combined with either Bioruptor or Bullet Blender resulted in the identification of 1582 (60%) and 1615 (62%) proteins, respectively. In contrast, using 8 M urea and Bioruptor for protein extraction resulted in 1022 proteins (39%), whereas employing Bullet Blender yielded 1446 proteins (55%). Sample amounts, ranging from 0.6 to 10 mg, were prepared with consistent protein extraction outcome obtained for samples ≥1.2 mg. Combining Ripa and 8 M urea with Bullet Blender increased protein identification to 2126 (81%). Proteins were classified by their cell components, molecular functions, and biological processes. Furthermore, a subclassification of proteins involved in the extracellular matrix (ECM) was introduced. We recommend the use of Ripa buffer, in combination with 8 M urea and Bullet Blender for extracting proteins from fresh-frozen human nerves weighing ≥1.2 mg.

## Introduction

Surgical repair or reconstruction of peripheral nerve injuries presents a clinical challenge, often yielding suboptimal outcomes. The nerve degeneration and regeneration processes are complex and involve activation of a variety of pathways necessary for converting the neurons and its related cells, such as the Schwann cells, from maintenance of the homeostatic conditions to production machinery after nerve injury. The intracellular processes involve upregulation of numerous genes associated with regeneration as well as subsequent changes in the proteomic patterns that are essential for regeneration and thereby for improving treatment strategies.^1,2^ Over time, regenerating axons may re-establish connections with their target tissues, leading to functional recovery, sometimes taking months to years.^3,4^ After that time, the “production machinery” returns to “maintenance” again, probably with subsequent changes in the proteomic pattern.

Proteomics has been used to study nerve degeneration, with a particular focus on Schwann cells,^5^ mainly in experimental models. However, our understanding of the changes occurring in humans after injury is limited.^6^ Injured peripheral nerves may exhibit changes in protein expression and composition, impacting the processes of inflammation, regeneration, extracellular matrix (ECM) remodeling, oxidative stress, neurotrophic support, myelin integrity, pain signaling, and cell signaling pathways.^7,8^ In humans, mass spectrometry has identified approximately 2617 distinct proteins in the terminal part of a healthy human posterior interosseous nerve in healthy individuals and in patients with type 1 and 2 diabetes.^9,10^ However, specific methodologies for effective protein extraction from peripheral nerve tissue, particularly in healthy, uninjured human nerves, remain underexplored. This gap in knowledge limits the understanding of peripheral nerve proteomics and the development of reliable approaches for studying nerve injury and repair, as well as neuropathy.

The limitations in the dynamic range and sensitivity of current proteomic techniques have resulted in the omission of many predicted protein products in experiments.^11^ These omitted proteins could provide insights for comprehending biological nerve processes and potentially open new paths of research.

Here, we report the findings obtained using different preparation methods and, through the analysis of varying amounts from a fresh frozen human sural nerve, all assessed using a data-dependent acquisition (DDA)-based label free quantification method for liquid chromatography–mass spectrometry (LC-MS). Our objective was to refine the protein extraction technique to achieve consistent outcomes with a minimal peripheral nerve tissue amount. This approach aimed to enable further investigations into peripheral nerve responses to injury and facilitate the subclassification of ECM proteins, while ensuring that differences in protein identification and yield arose from the methods used rather than biological variability.

## Experimental Section

### Subjects

Nerve tissue samples were obtained during a scheduled surgical procedure in which the sural nerve was harvested in connection with a nerve reconstruction procedure at the Department of Hand Surgery in Malmö, Sweden. The remaining portions of the harvested sural nerve, not needed for the nerve reconstruction procedure, were collected and promptly placed in a freezer and stored at −80 °C. Prior to surgery, an informed consent was retrieved from the donor.

### Ethical Approval

The study has been approved by the Regional Ethical Review Board in Lund, Sweden (no 311/2016). All study participants provided informed written consent. All procedures were carried out in line with relevant current guidelines and regulations.

### Sample Preparation

The fresh frozen human sural nerve samples, approximately 1 mg (1.5 mm^3^), were transferred to 1.5 mL of Axygentubes (maximum recovery). To the tissue samples, 100 μL extraction buffer was added, either 8 M urea in 100 mM ammonium bicarbonate/AMBIC (Method 1) or Ripa buffer (Sigma-Aldrich) (Method 2). The tubes were placed in a Bioruptor (Diagenode), 40 cycles (15 s on, 15 s off). Samples were centrifuged at 14,000*g* for 10 min. The supernatants were transferred to new tubes.

Fresh frozen human sural nerve pieces were transferred to Rino tubes containing the extraction buffers, 8 M urea, or Ripa buffer (Method 3 and Method 4) and placed in the Bullet Blender Storm Pro (BT24M, Next Advance, Inc. Troy, NY, USA), and the samples were run for 3 min at speed 8. Samples were centrifuged at 14,000*g* for 10 min. The supernatants were transferred to new tubes. The bullets in the Rino tubes were washed with 100 μL of 8 M urea or Ripa buffer followed by centrifugation, and the supernatants were added to the tubes containing the prior supernatants from the bullets.

**Figure 1:**
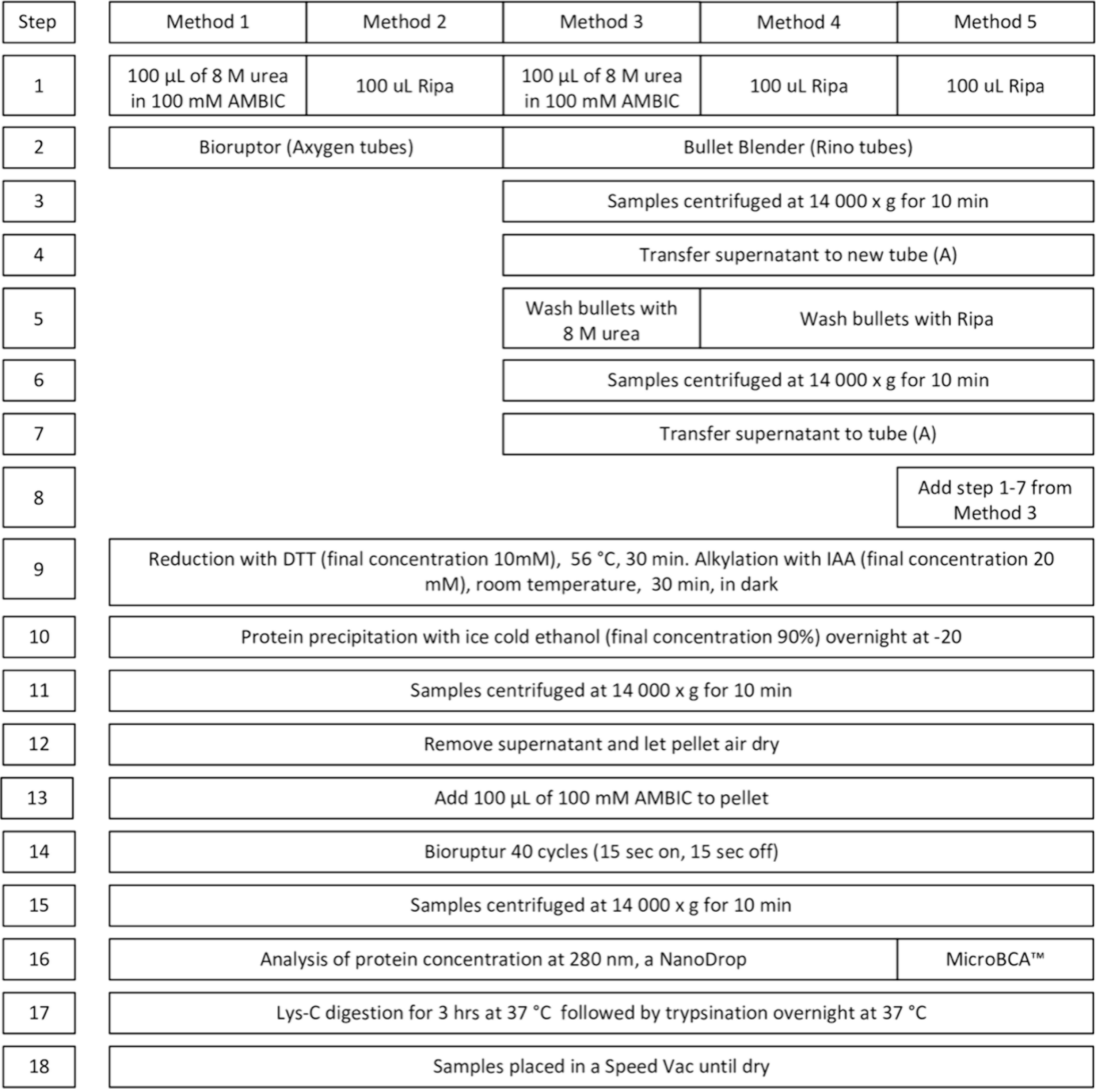
Workflow for sample preparation for Methods 1–5.

A final method (Method 5) was established by combining Methods 3 and 4. This method was used to establish the minimum amount of nerve tissue required to yield dependable results. Briefly, nerve tissue, ranging from 0.6–10 mg, was transferred to a Rino tube, and 100 μL of Ripa buffer was added and placed in the Bullet Blender as before. Samples were centrifuged at 14,000*g* for 10 min. The supernatants were transferred to a new Axygen tube. Bullets were washed with 100 μL of Ripa buffer and centrifuged, and the supernatant was pooled. Then, 100 μL of 8 M urea was added to the Rino tube and placed in Bullet Blender as before. Samples were centrifuged and supernatants was pooled to the tube with Ripa buffer supernatant. The bullets were washed with 8 M urea, centrifuged, and supernatant pooled with the earlier supernatants. An overview of the experimental workflow is given in Figure 1.

For this study, the primary aim was to evaluate the ability of the methods to maximize protein identifications rather than interreplicate variability, and as such, no technical replicates were performed for each method.

Protein concentration was measured at 280 nm using a NanoDrop (DeNovix DS-11, DeNovix Inc.; Wilmington, DE, USA) for Methods 1–4. The MicroBCA TM Protein Assay Kit (ThermoFisher Scientific) following the microplate procedure recommended by the manufacturer was used for samples prepared with Method 5.

The amount of 30 μg of protein was reduced with dithiothreitol (DTT) to a final concentration of 10 mM and heated at 56 °C for 30 min followed by alkylation with iodoacetamide (IAA) to a final concentration of 20 mM for 30 min at room temperature in the dark. Samples were then precipitated with ice cold ethanol to a final concentration of 90% overnight at −20 °C followed by centrifugation at 14,000*g* at 8 °C for 10 min. The pellets were resuspended with 100 μL of 100 mM AMBIC and sonicated using a Bioruptor (40 cycles (15 s on, 15 s off). Digestion was performed by adding LysC in a ratio of 1:50 (Lysyl Endopeptidase, Wako Mass Spectrometry grade 10 AU, Product number: 129–02541) for 3 h followed by adding trypsin in a ratio of 1:50 (Sequencing grade Modified Trypsin, Part No. V511A, Promega) to the samples and incubated overnight at 37 °C. The digestion was stopped by 10 μL of 10% trifluoroacetic acid (TFA). The samples were placed in a Speed Vac until they were dry. The samples were resolved in 30 μL of 2% acetonitrile (ACN) and 0.1% trifluoroacetic acid (TFA) prior to analysis in mass spectrometer.

### LC MS Analysis

The samples were analyzed on an Orbitrap Eclipse Tribrid mass spectrometer (Thermo Fischer Scientific, Waltham, MA) coupled with an Ultimate 3000 RSLCnano system (Thermo Fischer Scientific, Waltham, MA). A two-column setup was used on the HPLC system, and peptides were loaded into an Acclaim PepMap 100 C18 precolumn (75 μm × 2 cm, Thermo Scientific, Waltham, MA) and then separated on an EASY spray column (75 μm × 25 cm, nanoViper, C18, 2 μm, 100 Å) with the flow rate 300 nL/min. The column temperature was set at 45 °C. Solvent A (0.1% FA in water) and solvent B (0.1% FA in 80% ACN) were used to create a nonlinear gradient to elute the peptides. For the gradient, the percentage of solvent B was maintained at 2% for 4 min, increased from 2% to 25% for 100 min, and then increased to 40% for 20 min and then increased to 95% for 1 min and then kept at 95% for another 5 min to wash the column.

The samples were analyzed with the positive DDA mode. The full MS resolution was set to 120,000 at normal mass range and the AGC target was set to standard with the maximum injection time to auto. The full mass range was set at 350–1400 *m*/*z*. Precursors were isolated with an isolation window of 1.6 *m*/*z* and fragmented by HCD with a normalized collision energy of 30. MS2 was detected in the Orbitrap with a resolution of 15,000. The AGC target and the maximum injection time were set as standard and 50 ms, respectively.

#### Data Analysis

The raw DDA data were analyzed with Proteome Discoverer 2.5 Software (Thermo Fisher Scientific). Peptides were identified using SEQUEST HT against the UniProtKB human database (UP000005640_9606). The search was performed with the following parameters applied: static modification: cysteine carbamidomethylation; dynamic modifications: N-terminal acetylation and methionine oxidation. Precursor tolerance was set to 10 ppm, and fragment tolerance was set to 0.02 ppm. Up to 2 missed cleavages were allowed, and Percolator was used for peptide validation at a *q*-value of maximum 0.01. Extracted peptides were used to identify and quantify them by label-free relative quantification. The extracted chromatographic intensities were used to compare the peptide abundance across samples.

To classify proteins based on their cellular components, biological processes, and molecular functions, functional annotation was performed using Proteome Discoverer. The software utilizes Gene Ontology (GO) annotations to categorize proteins into these functional classes.

Proteome Discoverer assigns proteins to specific cellular components; nucleus, nonstructural extracellular, ECM, plasma membrane, other membranes, cytosol, cytoskeleton, mitochondria, ER/Golgi, translational apparatus, other cytoplasmic organelles, and other cell components. The biological processes in which the identified proteins are involved are divided into cell adhesion, cell–cell signaling, cell cycle or cell proliferation, cell death, cell organization and biogenesis, protein metabolism, DNA metabolism, RNA metabolism or transcription, other metabolic processes, stress response, transport, developmental processes, signal transduction, and other biological processes. The proteins are also categorized by their molecular functions, including signal transduction activity or receptor binding, transporter activity, enzyme regulatory activity, extracellular structural activity, bone, tooth, or skin structural activity, cytoskeletal activity, transcription regulatory activity, translation activity, chaperone-related activity, nucleic acid binding activity, kinase activity, and other molecular function.

In our data, the cell component of ECM, nonstructural extracellular, and the molecular function of extracellular structural activity were subclassified as presenting ECM proteins. The molecular functions of cytoskeletal activity and the biological process of cell adhesion and cell organization and biogenesis were also included in this subclassification.

#### Contamination of Keratins

To check for contamination of keratins in samples used in Method 5, a ratio was used between the abundance of identified keratins divided by the total abundance of all proteins in each sample.

#### Statistics

Data are presented as numbers. The chi-square test was used for nominal variables, for comparison of Method 1–5 protein identification to evaluate if there were significant associations between Method 1–5 and proteins identification. A *p*-value of <0.05 was considered statistically significant. Statistical calculations of characteristics are made using IBM SPSS Statistics version 26 for Mac (SPSS Inc.). The coefficient of variation (CV %) of replicates was calculated by evaluating the CV for proteins, precursors, and the proportion of missing data across replicates for each method.

## Results

### Buffer Impact on the Number of Protein Identification

**Figure 2:**
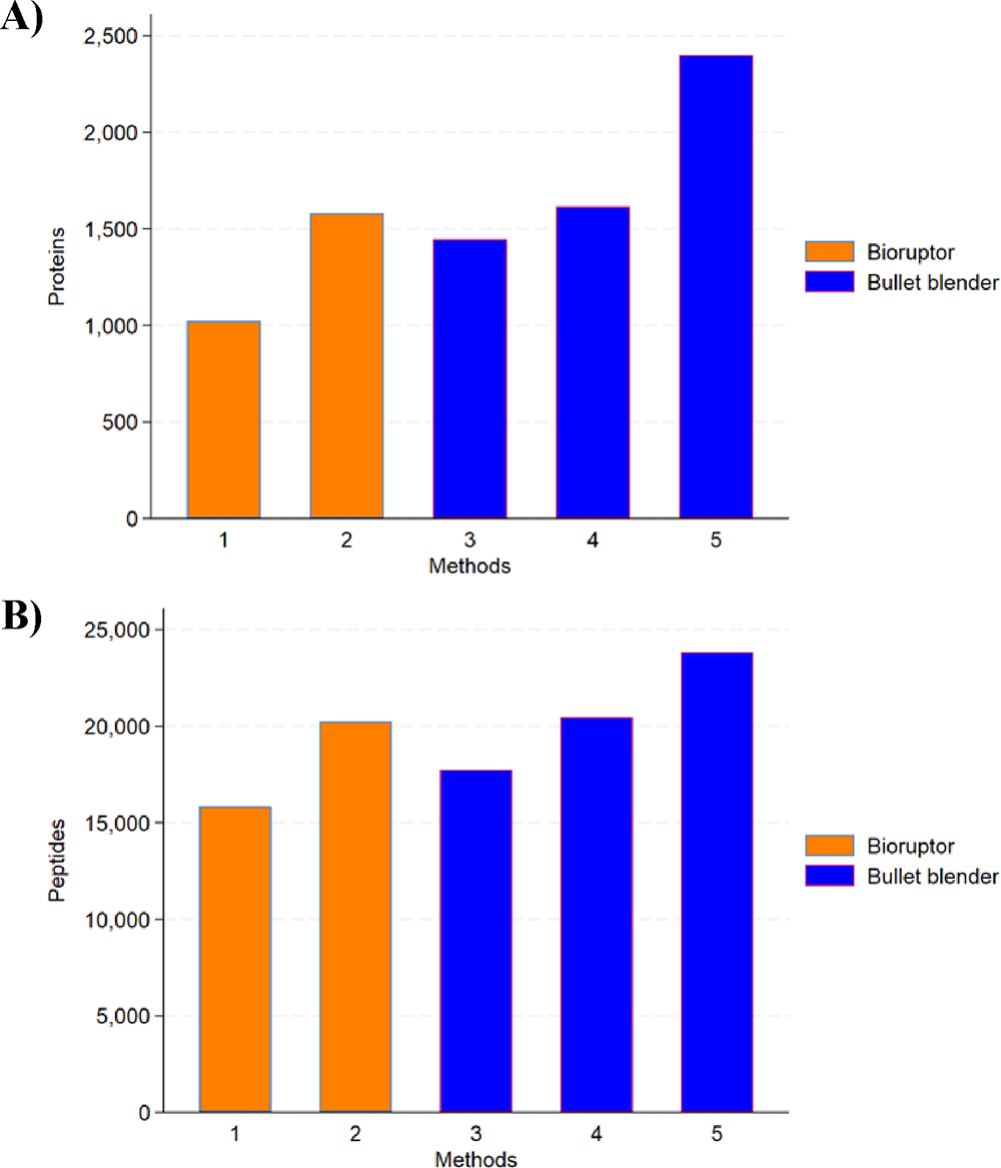
Comparison of (A) proteins and (B) peptides identified in Bioruptor or Bullet Blender lysed with urea and/or Ripa buffers for protein extraction from fresh frozen human sural nerve.

A significant difference was seen in the number of peptide and protein identified for samples lysed with 8 M urea in Methods 1 and 3 compared with the Ripa buffer in Methods 2 and 4. Of the total number of proteins (2619) discovered in this study with at least of 2 unique peptides identified, Methods 1 and 2 found 1022 and 1582 proteins, respectively (*p* < 0.001), while Methods 3 and 4 found 1446 and 1615 proteins, respectively (*p* < 0.001) (Figure 2, Supporting Information; Table S1).

When the two buffers, Ripa buffer and 8 M urea, were combined in Method 5, the number of proteins identified was 2126, being significantly higher than the protein identification of Methods 1–4 (*p* < 0.001) (Figure 2, Supporting Information; Table S2).

### Bioruptor and Bullet Blender Influence on Protein Extraction Efficiency

The potential impact of the homo-genization method on protein extraction efficiency was assessed by comparing the number of proteins identified in samples lysed with 8 M urea and Ripa buffer using either Bioruptor or Bullet Blender. The Bullet Blender showed significant advantage over Bioruptor in protein extraction with both 8 M urea and Ripa buffer. In combination with 8 M urea, the Bioruptor yielded 1022 proteins while with the Bullet Blender, 1446 proteins were identified (*p* < 0.001). In combination with Ripa buffer, the Bioruptor yielded 1582 proteins while with the Bullet Blender, 1615 proteins were identified (*p* < 0.001)) (Figure 2)

### Missed Cleavage

A low peptide missed cleavage rate was observed for Methods 1–4 (8.8%) for one missed cleavage and 0.4% for two missed cleavages. No difference was seen in the rate of missed cleavages between samples prepared using Method 5.

### Cellular Component, Molecular Function, and Biological Processes

A comparison of the extracted proteins and their cellular components was performed, showing that all the cellular components were more present in Ripa buffer compared to 8 M urea regardless of whether sample homogenizing was performed with Bioruptor or Bullet Blender. The proteins with cellular components adhering to nonstructural extracellular, plasma membrane, other membranes, other cytoplasmic organelles, other cell components, mitochondrion, ER/Golgi, and cytosol were more present when using Bullet Blender, while proteins including components for translational apparatus, ECM, nucleus, and cytoskeleton were more present when Bioruptor was used (Supporting Information; Table S3, Figure S1).

Proteins with molecular function of transporter activity, translational activity, signal transduction activity or receptor binding, other molecular function, nucleic acid binding activity, kinase activity, and enzyme regulator activity were more present in Ripa buffer compared to 8 M urea, while proteins with molecular function of extracellular structural activity and cytoskeletal activity were seen to be more present with 8 M urea (Supporting Information; Table S3).

Proteins adhering to all biological processes were more present in Ripa buffer than in 8 M urea.

Proteins with biological processes of transport, stress response, signal transduction, RNA metabolism or transcription, protein metabolism, other metabolic processes, other biological processes, developmental processes, cell–cell signaling, and cell adhesion were more present when using Bullet Blender, while proteins involved in the biological process of DNA metabolism, cell organization and biogenesis, and cell cycle or cell proliferation were equally present when applying the Bioruptor or Bullet Blender.

When combining Ripa buffer and 8 M urea in the extraction process, a clear increase in presence was seen in proteins adhering to all cellular components, molecular functions, and biological processes investigated except for the molecular function of extracellular structural activity and transporter activity, which were more present using Method 4 (Supporting Information; Figure S1, Table S3).

### The Minimum Amount for Protein Identification

**Figure 3:**
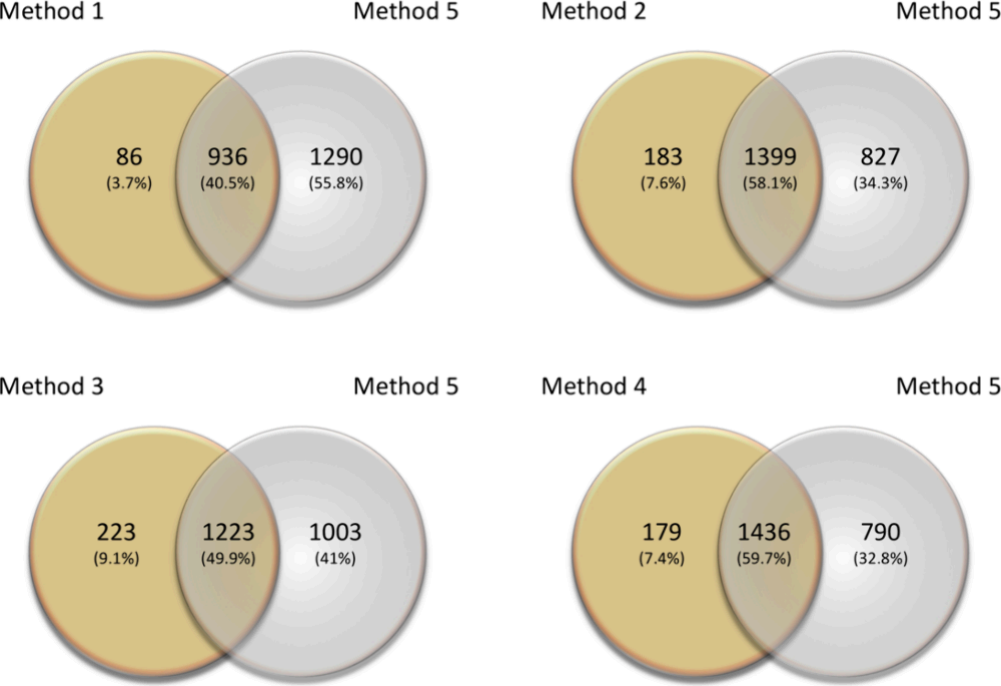
A Venn diagram showing overlaps of all identified proteins in Methods 1–4 compared to Method 5.

Different amounts, 0.6–10 mg, from the same sural nerve used for Methods 1–4, were prepared (Supporting Information, Table S2) and lysed with a two buffer approach, first, the Ripa buffer followed by 8 M urea and homogenized using the Bullet Blender (Method 5). All samples from size 1.2 to 10 mg showed a significant increase in the number of identified peptides and proteins compared to Methods 1–4 (*p* < 0.001) (Figure 3), while the smallest sample, 0.6 mg, showed the lowest number of identified peptides and proteins compared to the rest of the samples (1443 proteins compared to 1615 proteins identified in Method 4) (*p* = 0.28). This suggests a limit amount threshold for consistent protein extraction to lie between 0.6 and 1.2 mg (Supporting Information; Table S1). Checking for keratin contamination in the samples used in Method 5 showed that the lowest amount used (0.6 mg) had the highest ratio between keratin abundance and total protein abundance, 2.1 compared to 0.1–0.31% for the rest of the samples (Supporting Information; Figure S2)

### Investigation of Nerve Injury and Repair Pathways

With our objective to refine the protein extraction technique, a special focus was on whether the methods were successful in extracting specific proteins for further investigation of nerve injury and repair. The following pathways were selected due to their specific involvement in nerve injury and repair processes (Table 1). For the Schwann cell differentiation pathway (GO:0014037), the presence of 38% of the 29 unique proteins described in Uniprot was revealed. Similarly, for the ECM Organization (GO:0030198), 28% of the 215 unique proteins were found. For the Response to Reactive Oxygen Species pathway (GO:0000302), the identification of 34% of the 92 unique proteins was demonstrated. Myelination (GO:0042552) showed the presence of 26% of 101 unique proteins. Furthermore, the Cell Adhesion pathway (GO:0007155) revealed the identification of 23% of the 924 unique proteins. In the context of the Negative Regulation of Apoptotic Processes (GO:0043066) pathway, the identification of 26% of the 87 unique proteins was seen. For the Neurotrophin Signaling Pathway (GO:0048011), the identification of 14% of the 14 unique proteins was found. Lastly, for the Axon Guidance pathway (GO:0007411), 13% of the 228 unique proteins were identified (Table 1, Supporting Information; Table S4).

**Table 1:** Comparison of Protein Extraction Methods for Different Pathways, Indicating the Number of Reviewed Proteins from UniProt and Proteins Found in the Dataset for Methods 1–5

**pathway**	**GO Term**	**reviewed proteins on uniport/found in data set**	Method 1	Method 2	Method 3	Method 4	Method 5
**Schwann cell differentiation**	(GO:0014037)	29/11	6	8	12	7	11
**Neurotrophin Signaling Pathway**	(GO:0048011)	14/2	0	1	0	1	2
**Axon Guidance**	(GO:0007411)	228/29	12	19	16	21	25
**Extracellular Matrix Organization**	(GO:0030198)	215/60	40	46	46	46	52
**Response to Reactive Oxygen Species**	(GO:0000302)	92/32	18	25	24	25	27
**Myelination**	(GO:0042552)	101/26	12	17	16	19	24
**Cell Adhesion**	(GO:0007155)	924/211	106	145	134	149	174
**Negative Regulation of Apoptotic Processes**	(GO:0043066)	87/23	11	15	15	16	20

### The Coefficient of Variation (CV %) of Replicates

Method 5 showed the lowest missing data (8%) and a protein CV (15.3%). Method 1 had the lowest protein CV (12.1%) but exhibited the highest missing data (61%). Method 3 had a similar protein CV (11.9%) with 45% missing data, while Method 4 showed the highest protein CV (18.1%) and 38% missing data (Supporting Information; Table S5).

## Discussion

We investigated the influence of different extraction methods, comparing 8 M urea or Ripa buffers in combination with Bioruptor sonication or the Bullet Blender. In total, 2619 proteins were identified from freshly frozen human sural nerve samples harvested from the lower limb. The results are in accordance with a previously described study, where 2617 proteins were identified from the terminal branch of the posterior interosseus nerve tissue harvested from the upper limb from healthy individuals and patients with type 2 diabetes.^9^ When comparing samples extracted with Ripa buffer or 8 M urea, regardless of the homogenization method, the Ripa buffer was shown to be more efficient regarding the number of identified proteins. The Ripa buffer is known to be a versatile and effective lysis solution, well-suited for the extraction of a wide range of proteins encompassing whole cell, nuclear, mitochondrial, membrane receptors, cytoskeletal-associated, and soluble proteins.^12^ However, previous research has identified that many ECM proteins are insoluble in Ripa buffer but dissolve readily in urea buffer.^12^ Urea buffer has been found to be more preferentially enriched in the extracellular region compared to the Ripa buffer^12,13^ due to its ability to disrupt hydrogen bonding^14^ and other noncovalent interactions, effectively denaturing proteins and aiding in their solubilization. For Methods 1–4, extraction with 8 M urea showed the highest number of proteins of extracellular structural activity and cytoskeletal activity. Proteins from all other cell components as well as those involved in biological processes and molecular functions were more present in Ripa buffer (Supporting Information; Table S3). A two-step extraction approach that has shown promising results in other tissue types, such as tissue from three patients with breast tumors and a single subject with healthy breast tissue,^12^ was introduced in this study (Method 5). Its application in our study, where proteins from fresh frozen uninjured sural nerves were analyzed, yielded a significant increase in the number of identified proteins and peptides. The analysis of the coefficient of variation (CV%) for protein and precursor abundances as well as missing data provides a clearer picture of reproducibility and data completeness across the different methods. Method 5 exhibits the lowest percentage of missing data (8%), which suggests more reliable and comprehensive protein identification compared to the other methods. Although Method 1 shows the lowest protein CV (12.1%), indicating more consistent protein abundance across replicates, its high missing data (61%) limit its utility. Similarly, Method 3, with a CV of 11.9%, also demonstrates substantial missing data (45%). Method 4 has the highest protein CV (18.1%), indicating significant variability in protein abundance measurements, and has 38% missing data. While precursor CV values remain relatively consistent across methods, the variability in protein CV and missing data points to Method 5 as the most balanced approach, offering good reproducibility with comprehensive proteome coverage. These findings suggest that Method 5 aligns best with the study’s goal of maximizing protein identification.

We consistently observed better protein extraction using the Bullet Blender compared with Bioruptor in all of our methods. The Bullet Blender and Bioruptor have different mechanisms for tissue disruption. The Bullet Blender uses bead beating, while the Bioruptor uses sonication.

Previous research has suggested that missed cleavages might be sample- and method-dependent;^15^ in the present study, all 4 methods presented the same missed cleavage rate. When 8 M urea and Ripa buffers were combined (Method 5), the same missed cleavage rate was observed, indicating that the use of both buffers does not compromise the protein digestion process. In this study, Lys-c and trypsin was used for digestion, in order to reduce the abundance of missed cleaved peptides.^16^

Investigation of the minimum amount of tissue that could be used for protein extraction was made. The study introduces an amount-dependent factor in protein identification, revealing that the sample of 0.6 mg exhibited a limitation in identified peptides and proteins compared to samples ≥1.2 mg. The analysis of keratin contamination, a common issue in proteomic studies,^17^ also indicates that the smallest used amount was too low for reliable results. Since the study was performed in a common laboratory and not in a high-containment laboratory, contamination of keratins may cause a problem when samples are analyzed using mass spectrometry. Starting with a small amount of tissue might get higher contamination from the surroundings, leading to signal loss and decrease in protein identification. This finding might imply a threshold for consistent protein extraction, highlighting the importance of considering sample amount in experimental design to ensure robust and reproducible results.

We describe a protein extraction method that can be used for future investigation of the degeneration and regeneration processes occurring in injured peripheral nerves. Our method was shown to extract proteins associated with pathways relevant to nerve injury and regeneration including the ECM proteins. These proteins are relevant for promoting axonal outgrowth and Schwann cell migration in nerve regeneration.^1^ We identified a number of proteins from a fresh frozen human sural nerve. Some biological events, such as up-regulation of specific proteins related to eliciting a regenerative response in nerves, are rapidly induced in a peripheral nerve after injury,^18,19^ which should be considered when evaluating proteins in human nerves in relation to time after injury or harvest of specimens. However, the present fresh frozen sural nerves were not subjected to any injury other than during the harvest procedure during surgery; thus, the present nerve represents essentially an uninjured peripheral nerve.

Improving nerve regeneration is central after a nerve injury and subsequent repair or reconstruction because it directly addresses the restoration of neural connectivity, signal transmission, and functional integration within the peripheral nervous system. This in turn is essential for achieving optimal recovery and minimizing long-term impairments associated with nerve injuries.^20,21^ The pathway of axon guidance is fundamental for both the development and regeneration of axons after injury. Navigating regenerating axons toward their targets plays a key role in promoting neuronal survival, axonal outgrowth, and synaptic plasticity after a peripheral nerve injury, where, e.g., neurotrophin signaling pathways, including nerve growth factor (NGF) and brain-derived neurotrophic factor (BDNF), are involved in the process. While several of these neurotrophic factors already have been identified as important for nerve regeneration in experimental models, including infusion of the factors into nerve conduits after nerve reconstruction, revealing a superior retrograde neuroprotection as compared to reconstruction with nerve autografts across a 10 mm gap in the rat sciatic nerve,^22^ further understanding of these processes in humans is warranted. The Schwann cell differentiation pathway is decisive as it presents how the Schwann cell acquires its specialized structural and functional features important in supporting and interacting with the axons^23^ and their role in remyelination may be crucial for restoring nerve function.^24^ We included the pathways of reactive oxygen species, cell adhesion, and negative regulation of apoptotic processes due to their involvement in maintaining cellular homeostasis, mediating interaction between various cells and the ECM as well as their role in promoting repair. Further, the ECM undergoes significant changes after a nerve injury and during the nerve regeneration process.^25^ The pathway of ECM organization is important to include proteins involved in creating a permissive environment for axonal outgrowth and Schwann cell migration,^26^ where laminin, fibronectin, and type IV collagen play a critical role cell adhesion and migration. Laminin, fibronectin, tenascin, thrombospondin, and versican were all found in high abundances in all our methods. The ECM is highly tissue-specific and markedly heterogeneous.^27^ In this context, we propose a subclassification of ECM proteins that may be relevant in peripheral nerves and associated to regenerative events after nerve injury, which may not be fully captured by existing classification systems for different tissues.^28^ We find it essential to consider ECM proteins’ functional contributions beyond their structural roles, including their involvement in signaling, guidance, and regulation of cellular behavior. Additionally, to the cell components, ECM and nonstructural extracellular, as well as the molecular function, extracellular structural activity, we included the biological process of cytoskeletal activity in the ECM subclassification. Proteins with cytoskeletal activity contribute to structural support and to maintaining tissue integrity. Cytoskeletal proteins also play essential roles in growth cone formation, extension of the axons, and also their guidance during nerve regeneration.^29^ We also included the biological process of cell adhesion that includes molecules within the ECM that not only facilitate cell migration, axonal pathfinding, and interaction between regenerating axons and Schwann cells but also aid in maintaining tissue integrity and repair. Lastly, we included the molecular function of cell organization and biogenesis that includes ECM proteins that not only contribute to the formation of regenerative microenvironments, guiding axonal outgrowth, and facilitation of remyelination but also play crucial roles in regulating cellular activities and tissue homeostasis.^30^

## Conclusions

The findings of this study highlight the effectiveness of utilizing a protein extraction method that combines Ripa buffer, 8 M urea, and Bullet Blender for freshly frozen human sural nerves weighing ≥1.2 mg. By investigating the impact of different buffers, homogenization methods, and sample amounts on protein extraction efficiency, our study has developed a method for consistent protein extraction from freshly frozen uninjured human sural nerves from the lower limb. The detected proteins were classified by their cell components, molecular functions, and biological processes, and we introduce a subclassification of proteins involved in the ECM.

## Data Availability

Public access to research data on humans is restricted by the Swedish Authorities (Public Access to Information and Secrecy Act; https://www.government.se/information-material/2009/09/public-access-to-information-and-secrecy-act/), but data can be made available for researchers after a special review that includes approval of the research project by both the Swedish Ethical Review Authority (https://etikprovningsmyndigheten.se/en/) and the local authorities’ data safety committee in the health care sector in Region Skåne, Sweden; https://vardgivare.skane.se/kompetens-utveckling/forskning-inom-region-skane/utlamnande-av-patientdata-samradkvb/; “Samrådsgrupp för kvalitetsregister, vårddatabaser och beredning”; so-called KVB-group) – according to the “Law for ethical review of research on humans” [“Lag (2003:460) om etikprövning av forskning som avser människor”]. Data can only be available from the authors upon a reasonable request and after an appropriate application with subsequent written permission by the above-mentioned Swedish Ethical Review Authority as well as after approval by the mentioned health care sector in Region Skåne, Sweden (“Samrådsgrupp för kvalitetsregister, vårddatabaser och beredning”; so-called KVB-group).

## Supporting Information Available

Supplementary Table S1: summary table of proteins and peptides extracted from peripheral nerve tissue samples using the different methods; Method 1; 8 M urea and Bioruptor, Method 2; Ripa buffer and Bioruptor, Method 3; 8 M urea and Bullet Blender, and Method 4; Ripa buffer and Bullet Blender; Supplementary Table S2: table of proteins and peptides extracted from peripheral nerve tissue samples of different amount using Method 5; Ripa buffer followed by 8 M urea using Bullet Blender; Supplementary Table S3: number of proteins found categorized with specific cell component, biological process, and/or molecular function found with each method; Supplementary Table S4: details of Uniprot pathway search (the search for specific proteins defined by the Gene Ontology (GO database) was conducted on the 14th of February 2024 at Uniprot https://www.uniprot.org); Supplementary Figure S1: extraction of proteins with Methods 1–5 showing different distribution of cellular components between methods; Supplementary Figure S2: the ratio of keratin proteins to total proteins found in tissue samples of different amounts using Method 5 (PDF)
