# Supplementary material for: Refinement of Protein Extraction Protocols for Human Peripheral Nerve Tissue
Source: ACS Omega. 2025 Feb 2;10(5):5111–8. doi: 10.1021/acsomega.4c11373 (PMC11822717; doi:10.1021/acsomega.4c11373)

Supporting Information

Refinement of Protein Extraction Protocols for Human Peripheral Nerve Tissue

Drifa Frostadottir* [a,b], Charlotte Welinder[c], Raquel Perez [a,e], Lars B. Dahlin [a,b,d].

a) Department of Translational Medicine, Hand Surgery, Lund University, Malmö, Sweden
b) Department of Hand Surgery, Skåne University Hospital, Malmö, Sweden
c) Department of Clinical Sciences Lund, Mass Spectrometry, Lund University, Lund, Sweden.
d) Department of Biomedical and Clinical Sciences, Linköping University, Linköping, Sweden
e) Unit for Social Epidemiology, Department of Clinical Sciences Malmö, Lund University, Sweden

Contents

Supplementary Tables....................2
Supplementary Table S1: Summary table of proteins and peptides extracted from peripheral nerve tissue samples using the different methods....................2
Supplementary Table S2: Table of proteins and peptides extracted from peripheral nerve tissue samples of different amount. 2
Supplementary Table S3: Number of proteins found categorized with specific cell component, biological process and /or molecular function found with each method....................2
Supplementary Table S4: Uniprot pathways search details 4
Supplementary Table S5: The coefficient of variation (CV %) of replicates....................4
Supplementary Figures 5
Supplementary Figure S1: Extraction of proteins with Method 1-5 showing different distribution of cellular components between Methods....................5
Supplementary Figure S2: The ratio of keratin proteins to total proteins found in tissue samples of different amount using Method 5. 6
Supplementary Figure S3: The protein coverage % achieved with individual methods....................6

Supplementary Tables

Supplementary Table S1: Summary table of proteins and peptides extracted from peripheral nerve tissue samples using the different methods.

Method 1; 8 M urea and Bioruptor, Method 2; Ripa buffer and Bioruptor, Method 3; 8 M urea and Bullet Blender, and Method 4; Ripa buffer and Bullet Blender. P value of <0.05 was considered significant, with use of Chi-Square test. Significant p-values marked in bold.

Method 1		Method 2		P value	Method 3		Method 4		P value
Proteins	Peptides	Proteins	Peptides		Proteins	Peptides	Proteins	Peptides	
1022	15857	1582	20239	**<0.001**	1446	17737	1615	20455	**<0.001**

Supplementary Table S2: Table of proteins and peptides extracted from peripheral nerve tissue samples of different amount.

Method 5; Ripa buffer followed by 8 M urea using Bullet Blender. Significance testing using chi square to compare the number of proteins and peptides identified in Method 5 to those found in Method 4, as Method 4 yielded the highest number of proteins and peptides among Method 1-4. P value of < 0.05 was considered significant with use of Chi-Square test. Significant p-values marked in bold.

Method 5				
Sample	Amount in grams	Peptides	Proteins	P value
Total Median		23028	2126	**<0.001**
a	0.0006	19601	1443	0.279
b	0.0012	22901	2116	**<0.001**
c	0.0022	21784	1822	**<0.001**
d	0.0027	22919	2014	**<0.001**
e	0.0034	22868	2104	**<0.001**
f	0.0047	22705	2068	**<0.001**
g	0.0055	23003	2118	**<0.001**
e	0.0065	23032	2226	**<0.001**
i	0.0086	22621	2014	**<0.001**
j	0.0099	22746	2072	**<0.001**

Supplementary Table S3: Number of proteins found categorized with specific cell component, biological process and /or molecular function found with each method.
Bold represents the highest number found for Method 1-5. The highest number found for method 1-4 is marked with *. Methods with homogenization with Bioruptor is presented in orange. Methods with homogenization with Bullet Blender are presented in blue

Total protein found 2619	Method 1<br>1022	Method 2<br>1582	Method 3<br>1446	Method 4<br>1615	Method 5<br>2126
**Extracellular matrix proteins**					
Extracellular matrix (Cell component)	75	84 *	83	81	**97**
Non-structural extracellular (Cell component)	323	412	403	444 *	**522**
Extracellular structural activity (Molecular function)	43	44	**47 ***	40	46
Cytoskeletal activity (Molecular function)	176	234	245 *	224	**333**
Cell adhesion (Biological process)	95	136	121	141*	**188**
Cell organization and biogenesis (Biological process)	426	667 *	620	667 *	**1017**
**Cell components**					
Translational apparatus	64	86 *	82	73	**97**
Plasma membrane	356	529	462	553 *	**775**
Other membranes	468	734	630	744 *	**1145**
Other cytoplasmic organelle	167	231	220	256 *	**370**
Other cell components	768	1180	1095	1211 *	**1781**
Nucleus	418	660 *	612	643	**1012**
Mitochondrion	121	215	181	226 *	**352**
ER/Golgi	254	379	320	397 *	**587**
Cytosol	549	860	775	871 *	**1292**
Cytoskeleton	247	350 *	340	336	**505**
**Molecular Functions**					
Transporter activity	58	84	65	**87 ***	84
Translation activity	17	23 *	19	22	**81**
Signal transduction activity or receptor binding	148	208	184	215 *	**277**
Other molecular function	840	1310	1184	1349 *	**1967**

Nucleic acid binding activity	158	246 *	233	232		**366**
Kinase activity	17	34 *	27	41		**89**
Enzyme regulator activity	112	165	156	168		**238**
**Biological processes**						
Transport	283	406	377	420 *		**606**
Stress response	285	440	373	452 *		**701**
Signal transduction	226	332	303	347 *		**541**
RNA metabolism or transcription	83	113	118	125 *		**218**
Protein metabolism	282	436	392	439 *		**680**
Other metabolic processes	524	860	750	879 *		**1350**
Other biological processes	812	1201	1117	1222 *		**1774**
DNA metabolism	33	54 *	54 *	54 *		**89**
Developmental processes	267	391	358	398 *		**578**
Cell-cell signaling	50	74	66	80 *		**110**
Cell cycle or cell proliferation	164	247 *	231	247 *		**395**

Supplementary Table S4: Uniprot pathways search details

The search for specific proteins defined by the Gene Ontology (GO database) was conducted on the 14th of February 2024 at Uniprot https://www.uniprot.org.

Search word	Reviewed (Swiss-prot) Proteins found
"Schwann cell differentiation GO:0014037"	29
“Extracellular matrix Organization GO:0030198”	214
“Response to Reactive Oxygen Species GO:0000302”	92
“Myelination GO:0042552”	101
“Cell Adhesion GO:0007155”	924
“Negative Regulation of Apoptotic Processes GO:0043066”	87
“Neurotrophin Signaling GO:0048011”	14
“Axon Guidance GO:0007411”	228

Supplementary Table S5: The coefficient of variation (CV %) of replicates
The CV% was calculated by evaluating the CV for proteins, precursors, and the proportion of missing data across replicates for each method

Method	Protein CV	Precursor CV	% Missing
1	12,106	1,457	61%
2	16,349	1,477	40%
3	11,917	1,562	45%
4	18,093	1,475	38%
5	15,330	1,605	8%

Supplementary Figures

Supplementary Figure S1: Extraction of proteins with Method 1-5 showing different distribution of cellular components between Methods.

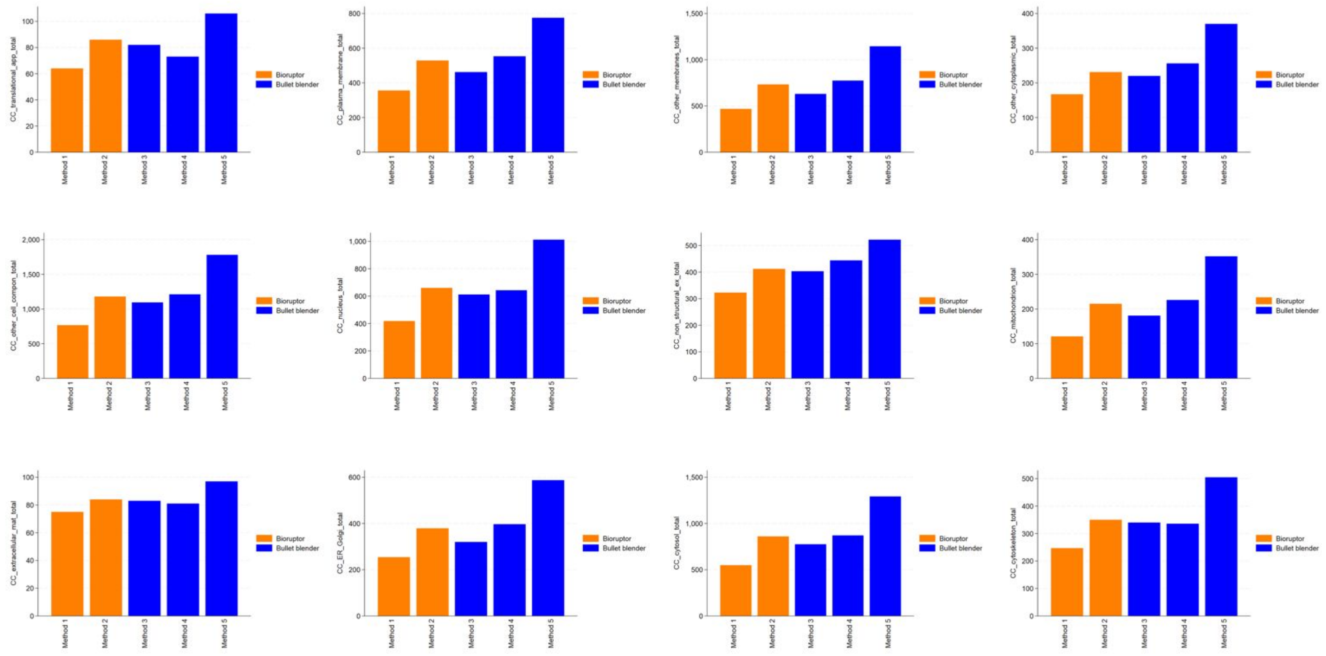

Supplementary Figure S2: The ratio of keratin proteins to total proteins found in tissue samples of different amount using Method 5.

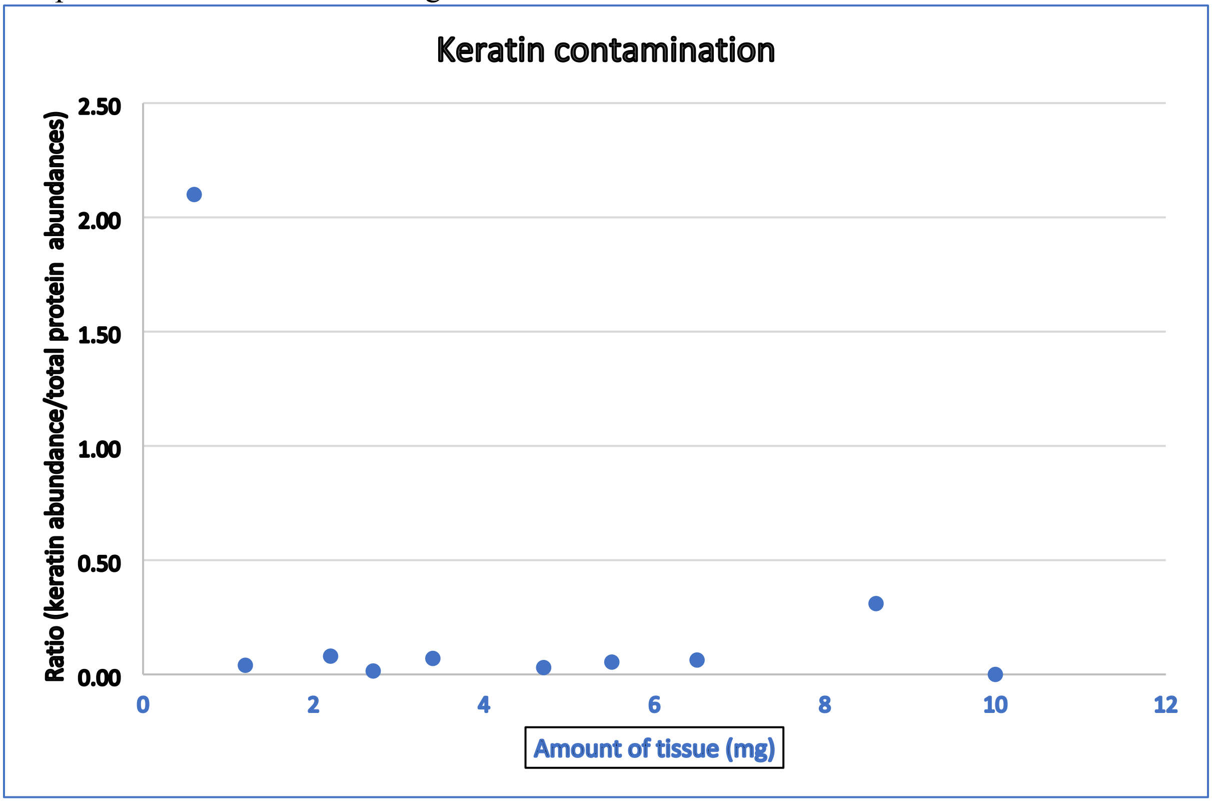

Supplementary Figure S3: The protein coverage % achieved with individual methods.

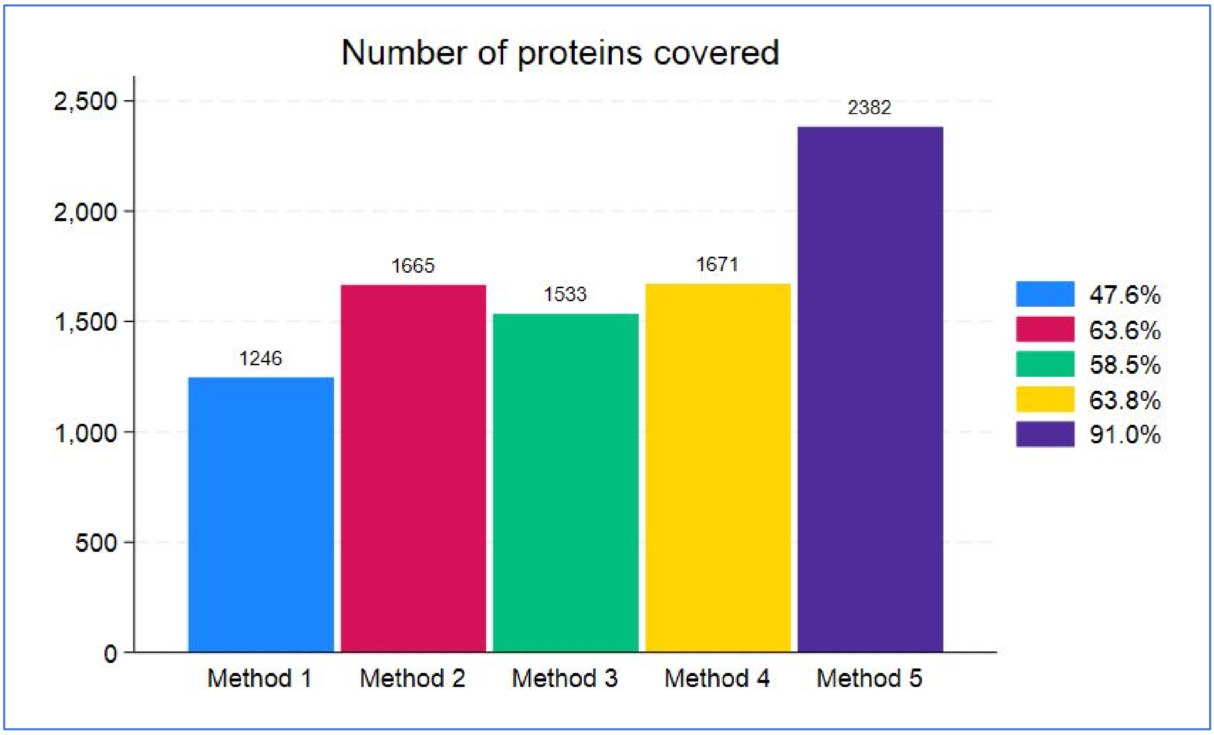

Supplement: Supplementary file 1 — ao4c11373_si_001.pdf [file ao4c11373_si_001.pdf]
